# Pharmacodynamics of Aerosolized Fosfomycin and Amikacin against Resistant Clinical Isolates of Pseudomonas aeruginosa and Klebsiella pneumoniae in a Hollow-Fiber Infection Model: Experimental Basis for Combination Therapy

**DOI:** 10.1128/AAC.01763-16

**Published:** 2016-12-27

**Authors:** Fekade Bruck Sime, Adam Johnson, Sarah Whalley, Anahi Santoyo-Castelazo, A. Bruce Montgomery, Kathie Ann Walters, Jeffrey Lipman, William W. Hope, Jason A. Roberts

**Affiliations:** a Centre for Translational Anti-infective Pharmacodynamics, School of Pharmacy, The University of Queensland, Brisbane, Queensland, Australia; b Burns, Trauma and Critical Care Research Centre, The University of Queensland, Brisbane, Queensland, Australia; c Department of Molecular and Clinical Pharmacology, The University of Liverpool, Liverpool, United Kingdom; d Cardeas Pharma Corporation, Seattle, Washington, USA

**Keywords:** pharmacokinetics, pharmacodynamics, multidrug resistance, nebulized

## Abstract

There has been a resurgence of interest in aerosolization of antibiotics for treatment of patients with severe pneumonia caused by multidrug-resistant pathogens. A combination formulation of amikacin-fosfomycin is currently undergoing clinical testing although the exposure-response relationships of these drugs have not been fully characterized. The aim of this study was to describe the individual and combined antibacterial effects of simulated epithelial lining fluid exposures of aerosolized amikacin and fosfomycin against resistant clinical isolates of Pseudomonas aeruginosa (MICs of 16 mg/liter and 64 mg/liter) and Klebsiella pneumoniae (MICs of 2 mg/liter and 64 mg/liter) using a dynamic hollow-fiber infection model over 7 days. Targeted peak concentrations of 300 mg/liter amikacin and/or 1,200 mg/liter fosfomycin as a 12-hourly dosing regimens were used. Quantitative cultures were performed to describe changes in concentrations of the total and resistant bacterial populations. The targeted starting inoculum was 10^8^ CFU/ml for both strains. We observed that neither amikacin nor fosfomycin monotherapy was bactericidal against P. aeruginosa while both were associated with rapid amplification of resistant P. aeruginosa strains (about 10^8^ to 10^9^ CFU/ml within 24 to 48 h). For K. pneumoniae, amikacin but not fosfomycin was bactericidal. When both drugs were combined, a rapid killing was observed for P. aeruginosa and K. pneumoniae (6-log kill within 24 h). Furthermore, the combination of amikacin and fosfomycin effectively suppressed growth of resistant strains of P. aeruginosa and K. pneumoniae. In conclusion, the combination of amikacin and fosfomycin was effective at maximizing bacterial killing and suppressing emergence of resistance against these clinical isolates.

## INTRODUCTION

For patients with pneumonia, the epithelial lining fluid (ELF) is an important subcompartment for a complete understanding of drug exposure-response relationships. Several factors can affect penetration of a systemically administered antibiotic into the ELF, including the blood-bronchus barrier, drug protein binding, physicochemical properties of the drug (hydrophilic-lipophilic balance and molecular size), pH at site of infection, and the degree of ionization of drug molecules (1, 2). While substantial penetration into the ELF has been observed for some antibiotics, for many others the concentrations achieved in the ELF may be subtherapeutic, particularly when the MIC for the etiologic organism is high, as commonly observed with pathogens like Pseudomonas aeruginosa (2) which commonly cause severe pneumonia. Hydrophilic antibiotics such as aminoglycosides are considered to poorly penetrate into the ELF and have a low probability of achieving therapeutic concentrations with systemic administration (2). Consequently, there is a high risk of clinical failure as well as of the emergence of resistant bacteria when pathogenic bacteria are exposed to suboptimal concentrations of antibiotic.

The inhalation route of administration may enable the delivery of effective antibiotic concentrations into the lungs (3). Given the strong relationship between antibiotic exposure and bacterial killing as described in *in vitro* models, optimizing concentrations at the site of infection may lead to improved clinical effects. A number of antibiotics have now been specifically formulated into aerosolized dosage forms although pharmacokinetic/pharmacodynamic (PK/PD) studies to confirm the microbiological effects of the high concentrations of antibiotics associated with this mode of administration remain sparse (4). One promising therapeutic option is a combination amikacin-fosfomycin product, which is currently being tested for clinical effectiveness (ClinicalTrials.gov identifier NCT02218359 [https://clinicaltrials.gov/ct2/show/NCT02218359]). It is administered via an investigational eFlow inline nebulizer system in mechanically ventilated patients. While promising results have been reported in early-phase clinical studies (5), the pharmacodynamic interaction of the combination regimen with the inhalational dose for clinical use is yet to be fully elucidated.

The aim of this study was to describe the individual and combined antibacterial effects (bacterial killing and suppression of emergence of resistance) of simulated epithelial lining fluid exposures of amikacin and fosfomycin when they are administered by the inhalational route against clinical isolates of P. aeruginosa and Klebsiella pneumoniae.

## RESULTS

### *In vitro* susceptibility.

Results of the susceptibility testing by the EUCAST and CLSI methods were similar. The MICs of amikacin for the P. aeruginosa clinical isolate (PA SAT 290) and the K. pneumoniae clinical isolate (K.p. 301) were 16 and 2 mg/liter, respectively, whereas the MIC of fosfomycin for both clinical isolates was 64 mg/liter.

### Mutation frequency.

The mutational frequencies of P. aeruginosa clinical isolate PA SAT 290 at a concentration of four times the MIC were 2.37 × 10^−6^ against fosfomycin and <1.32 × 10^−8^ for amikacin alone or for amikacin in combination with fosfomycin. The mutational frequencies of K. pneumoniae clinical isolate K.p. 301 at four times the MIC were 6.96 × 10^−6^, 2.00 × 10^−5^, and <8.7 × 10^−10^ against fosfomycin, amikacin, and the combination of amikacin and fosfomycin, respectively.

### HFIM.

**FIG 1:**
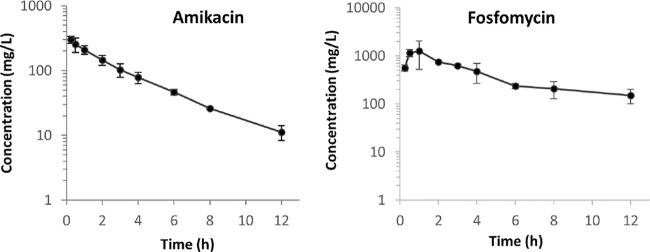
Observed concentration-time profiles of amikacin and fosfomycin during the first dosing interval.

**FIG 2:**
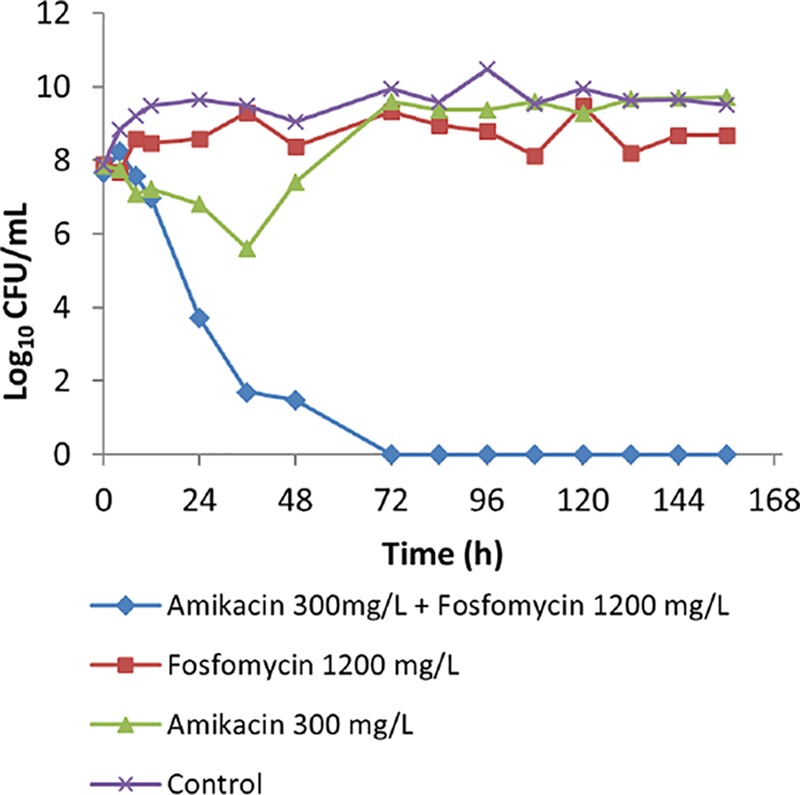
The effect of amikacin-fosfomycin combination therapy on the density of the total bacterial population of a P. aeruginosa clinical isolate (PA SAT 290) in an HFIM simulating pharmacokinetic decay of peak concentrations of 300 mg/liter amikacin–1,200 mg/liter fosfomycin in comparison with growth with monotherapy with each antibiotic.

The measured concentrations of amikacin and fosfomycin were close to expected concentrations considering linear pharmacokinetic decay, with half-lives of 3 h and 6 h, respectively (Fig. 1). Figure 2 summarizes the results of a hollow-fiber infection model (HFIM) investigation of the effect of amikacin-fosfomycin combination therapy on the total bacterial population of PA SAT 290 (P. aeruginosa) in comparison with monotherapy with each drug. Amikacin monotherapy showed only transient initial bacterial killing (about 2 logs) with rapid regrowth within 72 h such that the total bacterial concentration was comparable to that of the control arm with no drug treatment. Fosfomycin monotherapy, on the other hand, did not show any bacterial killing during the entire course of treatment. In contrast, treatment with the combination of amikacin and fosfomycin resulted in rapid bacterial killing with complete eradication of the bacterial population within 72 h. The bactericidal activity of the combination was sustained throughout the 7-day experiment with no regrowth.

**FIG 3:**
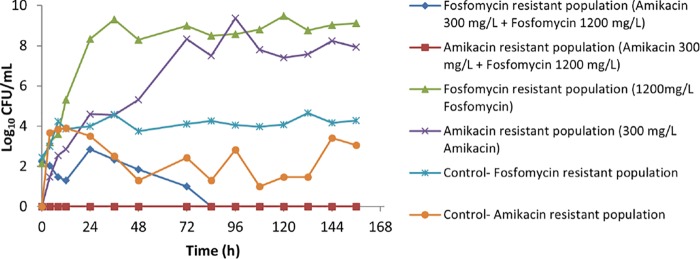
The effect of amikacin-fosfomycin combination therapy on the density of resistant bacterial populations of a P. aeruginosa clinical isolate (PA SAT 290) in an HFIM simulating pharmacokinetic decay of peak concentrations of 300 mg/liter amikacin–1,200 mg/liter fosfomycin in comparison with growth with monotherapy with each antibiotic.

The effect of the amikacin-fosfomycin combination on the resistant bacterial population of PA SAT 290 (P. aeruginosa) is illustrated in Fig. 3. Monotherapy with each drug resulted in rapid growth of resistant strains, which increased to a density of approximately 10^8^ to 10^9^ CFU/ml within 24 to 48 h. No growth of amikacin-resistant strains was observed after the combination therapy, whereas in the control arm growth of amikacin-resistant strains was observed throughout the 7 days of treatment. Similarly, there was a steady population of fosfomycin-resistant strains in the control arm (approximately 10^4^ CFU/ml) over the 7-day experiment. However, the combination therapy was effective in suppressing the growth of fosfomycin-resistant strains, resulting in complete eradication after 72 h.

**FIG 4:**
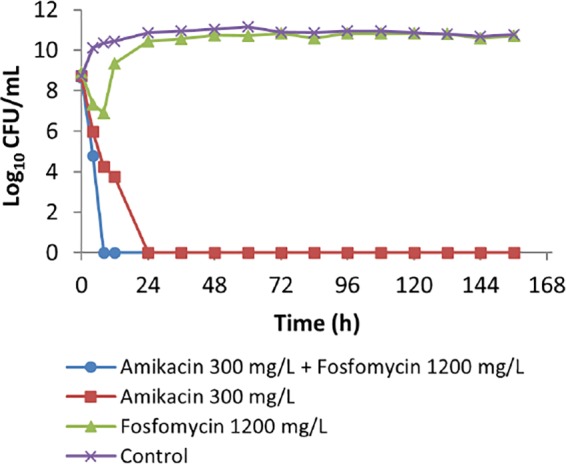
The effect of amikacin-fosfomycin combination therapy on the density of the total bacterial population of a K. pneumoniae clinical isolate (K.p. 301) in an HFIM simulating pharmacokinetic decay of peak concentrations of 300 mg/liter amikacin–1,200 mg/liter fosfomycin in comparison with growth with monotherapy with each antibiotic.

**FIG 5:**
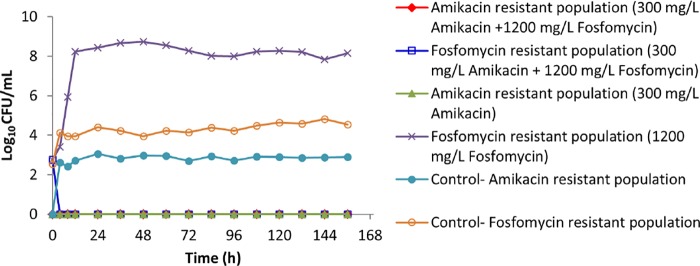
The effect of amikacin-fosfomycin combination therapy on the density of resistant bacterial populations of a K. pneumoniae clinical isolate (K.p. 301) in an HFIM simulating pharmacokinetic decay of peak concentrations of 300 mg/liter amikacin–1,200 mg/liter fosfomycin in comparison with growth with monotherapy with each antibiotic.

Figure 4 shows the effect of amikacin and/or fosfomycin therapy on total bacterial density of the K. pneumoniae clinical isolate (K.p. 301). Monotherapy with amikacin was effective in achieving maximal bacterial killing, unlike monotherapy with fosfomycin, which showed only transient initial killing followed by rapid regrowth that plateaued at a bacterial density comparable to that of the untreated control (Fig. 4). The combination therapy with similar target amikacin-fosfomycin peak concentrations resulted in faster bacterial killing. Further examination of the effect of the combination therapy on the resistant population (Fig. 5) shows no growth of resistant subpopulations that existed at baseline. However, fosfomycin monotherapy was associated with a rapid increase in the density of a fosfomycin-resistant population, peaking within 12 h, while in the untreated control there was no increase in the fosfomycin-resistant population from the baseline level.

## DISCUSSION

Considering the EUCAST clinical susceptibility breakpoints of conventional systemic (intravenous) dosing regimens of amikacin and fosfomycin, neither of the clinical isolates investigated in this study was susceptible to either antibiotic with the exception of the K. pneumoniae isolate, which was susceptible to amikacin. Thus, the infections caused by these nonsusceptible pathogens would not be considered treatable by conventional systemic therapy with either amikacin or fosfomycin. An inhalational mode of administration may enable the achievement of bactericidal concentrations at the site of infection and act as an adjunct to coadministered systematic antibiotic therapy (4, 6). High concentrations of amikacin and fosfomycin can be achieved in the epithelial lining fluid by this mode of drug administration while systemic exposure remains minimal, thereby potentially avoiding well-described toxicities (5, 7). In the current study, we assessed the pharmacodynamics of simulated epithelial lining fluid concentrations of amikacin and fosfomycin, alone and in combination, to describe effect on bacterial killing and suppression of resistance.

Our results suggest that the amikacin-fosfomycin combination achieves improved bacterial killing against P. aeruginosa compared to that with monotherapy (Fig. 2). In comparison, fosfomycin monotherapy had little discernible effect. For amikacin monotherapy, although initial bacterial killing at 12 h was similar to that of the combination, later bacterial killing was significantly lower than that observed with the combination. Similarly, more rapid bacterial killing was observed with the combination than with the monotherapies against the K. pneumoniae isolate (Fig. 4). Indeed, both monotherapies showed greater effects than those against the P. aeruginosa isolate, with fosfomycin showing modest bacterial killing in the first 8 h, after which there were no obvious effects. Amikacin alone was more significantly bactericidal than fosfomycin and then suppressed growth of the K. pneumoniae for the duration of the experiment.

Although the exact molecular mechanisms are unknown, the pharmacodynamic interaction between fosfomycin and an aminoglycoside antibiotic may occur due to enhanced access for the aminoglycoside to the target site within the bacterial ribosome. Aminoglycosides, which are hydrophilic molecules, may have a submaximal distribution across the cell wall into their binding sites in the cytosol (ribosomes) that could be a limiting factor for their antibacterial action and an important mechanism of resistance (8). Fosfomycin works by interfering with an initial step in the synthesis of building blocks of the bacterial cell wall, thereby disrupting the integrity of this important barrier (9). Consequently, it may enhance penetration of aminoglycoside drug molecules into the bacterial cells. For example, MacLeod et al. (10) demonstrated that fosfomycin enhances uptake of a radiolabeled [^3^H]tobramycin in a dose-dependent manner by interfering with either active or passive uptake processes in P. aeruginosa. Consistent with our findings for amikacin against P. aeruginosa (Fig. 2), the authors also demonstrated that enhanced bacterial killing by tobramycin was observed when it was combined with fosfomycin.

In addition to improved bacterial killing, the current study showed that the combination effectively prevented emergence of the resistant subpopulations observed with either amikacin or fosfomycin alone against the P. aeruginosa isolate (Fig. 3). For the K. pneumoniae isolate, no growth of an amikacin-resistant subpopulation was observed after monotherapy (Fig. 5), while there was a steady subpopulation of resistant strains in the control treatment arm. Given that the K. pneumoniae isolate was generally susceptible to amikacin and that amikacin exhibits concentration-dependent killing, it is likely that the relatively high concentration (300 mg/liter) resulted in a bactericidal effect against these less susceptible subpopulations. For fosfomycin on the other hand, monotherapy resulted in rapid growth of resistant populations, consistent with previous reports of its use against Gram-negative bacteria (11–13). However, when fosfomycin was combined with amikacin, the fosfomycin-resistant subpopulations were completely eradicated.

This study has several limitations. First, it fails to account for the host immune response, in the presence of which the exposure-response relationship may be different. Nonetheless, HFIM is considered highly predictive of clinical microbiological outcomes. Another important limitation is that only two clinical isolates were investigated, and the number of observations per treatment arm was limited due to the high cost of running the HFIM. Further, given the lack of data on the airway pharmacokinetics of the study antibiotics, the simulated concentrations were based on the assumption of similar pharmacokinetic decay patterns observed with systemic therapy. Thus, the simulated concentrations provide only a gross approximation of airway exposure given the current understanding of the study drugs' pharmacokinetics.

In conclusion, the findings of this study support the aerosolization of the amikacin-fosfomycin combination to enable high drug concentrations in the airway so as to rapidly decrease bacterial burden and prevent the emergence of resistant subpopulations, particularly against highly resistant infections. Further, the results demonstrate that monotherapy with either amikacin or fosfomycin can risk emergence of resistant subpopulations, which can be effectively suppressed by the combination of these antibiotics. It therefore appears advantageous to use the amikacin-fosfomycin combination to maximize bacterial killing and suppress the emergence of resistance.

## MATERIALS AND METHODS

### Antimicrobial agents.

For *in vitro* susceptibility testing, analytical reference standards of amikacin and fosfomycin were used. For hollow-fiber infection model (HFIM) studies, amikacin solution (pH 7.0; 100 mg/ml amikacin base, 3-ml ampoules) and fosfomycin solution (pH 8.0; 40 mg/ml fosfomycin base, 3-ml ampoules) were supplied by Cardeas Pharma and used undiluted by taking an appropriate volume corresponding to the required dose of simulated peak concentrations. Fosfomycin ampoules were stored in a refrigerator until just before dosing when the required volume for injection was transferred into a sterile delivery syringe in a biosafety cabinet. Similarly, amikacin ampoules were stored in the original package at room temperature, and just before dosing, the required volume was aspirated into the delivery sterile syringe in a biosafety cabinet.

### Bacterial isolates.

A clinical isolate of P. aeruginosa (PA SAT 290) sourced from patients with ventilator-associated pneumonia as part of an ongoing therapeutic trial was supplied by Cardeas Pharma. A clinical isolate of K. pneumoniae (K.p. 301) was obtained from the Antimicrobial Pharmacodynamics and Therapeutics Group, Department of Molecular and Clinical Pharmacology, the University of Liverpool. All bacteria were stored in cation-adjusted Mueller-Hinton II broth (CA-MH) containing 10% glycerol in a freezer at −80°C. Prior to each experiment, fresh isolates were grown on Mueller-Hinton agar plates incubated at 37°C for 24 h and used for the preparation of all inocula. For the hollow-fiber studies, the bacterial suspension for inoculation was prepared by taking five colonies of bacteria from the freshly grown agar plates and incubating them in 40 ml of CA-MH broth at 37°C for 24 h with constant agitation. Based on prior growth curve analysis, these amounts would give an inoculum concentration of about 1 × 10^8^ CFU/ml. The concentrations of the bacterial suspension in the final inoculum were confirmed by quantitative cultures.

### *In vitro* susceptibility testing.

The broth microdilution method was used for determination of the MIC for the organisms in accordance with the recommendations of the Clinical and Laboratory Standards Institute (CLSI) and European Committee on Antimicrobial Susceptibility Testing EUCAST (14, 15). In brief, serial 2-fold dilutions of each antibiotic were prepared in CA-MH broth and aliquoted into round-bottom microtiter plates (CLSI tests) and flat-bottom microtiter plates (EUCAST test). A standardized inoculum suspension prepared in CA-MH broth was then added to yield approximately 5 × 10^5^ CFU/ml. For fosfomycin susceptibility tests, all of the CA-MH broth used was supplemented with 25 mg/liter glucose-6-phosphate. The inoculated plates were then incubated at 37°C for 16 to 20 h. The optical density of each well in the flat-bottom microtiter plates was read using EUCAST MIC read software coupled to a Multiskan FC Microplate Photometer (Thermo Scientific). The lowest concentration of the antibiotic that resulted in a blank-subtracted optical density value of less than 0.1 times that of the positive growth control was identified as the MIC_90_. For the round-bottom microtiter plates, the lowest concentration of the antibiotic that completely inhibited growth was visually identified as the MIC in accordance with the CLSI recommendations. MIC tests were run in 10 replicates (five replicates for each method) on two different occasions; the mode of the determined values was reported as the MIC.

### Mutation frequency (MF).

To assess the frequency of mutation that leads to emergence of resistance, first an initial inoculum of 10^2^ CFU/ml was incubated in CA-MH broth for 24 h at 37°C. Next, the total bacterial concentration was estimated by plating on drug-free CA-MH agar plates and drug-containing CA-MH agar plates (at 4× MIC). The bacterial concentration was determined by quantitative culture, and the drug resistance frequency was estimated from the ratio of growth found on the drug-containing plates after 48 h of incubation to that of the starting inoculum.

### HFIM.

The circuit system for the hollow-fiber infection (HFIM) model was set up as previously described (16). FiberCell Systems cartridge C2011 was used for all experiments. In the experiments investigating the amikacin-fosfomycin combination, a supplementing compartment was introduced to simulate the differential clearance of the two antibiotics in accordance with the method described by Blaser (17). The CA-MH broth used in all experiments was supplemented with 25 mg/liter glucose-6-phosphate.

Given the lack of data on the airway pharmacokinetics of the study drugs, simulated concentration-time profiles were based on assumed half-lives inferred from systemic therapy (3 h and 6 h for amikacin and fosfomycin, respectively) (18, 19). Since very high concentrations of amikacin and fosfomycin can be achieved with aerosolized administration (5, 7), with potential mucin antagonism (∼95%) and a low to moderate exposure from this mode of delivery, the effects of monotherapy with amikacin or fosfomycin against the P. aeruginosa and K. pneumoniae clinical isolates were investigated at the target peak concentrations of 300 mg/liter amikacin and 1,200 mg/liter fosfomycin. The antibacterial effect of the combination was also studied using initial concentrations of 300 mg/liter amikacin plus 1,200 mg/liter of fosfomycin. In all of the HFIM studies, the antibiotics were dosed every 12 h as a bolus infusion for a 7-day duration. Control experiments with no drug treatment were run simultaneously under the same conditions as used for the treatment arms. Quantitative cultures were performed on bacterial samples taken from the extracapillary space of the hollow-fiber cartridge to determine the concentrations of the total bacteria and of the resistant population. For these analyses, serial samples were taken on day 1 before administration of the first drug(s) dose at 4 h, 8 h, and 12 h and subsequently before administration of each dose for the rest of the 7-day experiment. Samples were washed with sterile phosphate-buffered saline, and the concentrations of the total bacterial population and of the drug-resistant subpopulation were determined by plating on drug-free and drug-containing CA-MH agar plates (at four times the MIC of the bacteria).

### Drug assay.

Fosfomycin concentrations were measured using a validated ultrahigh-performance liquid chromatography tandem mass spectrometry method. Briefly, fosfomycin was extracted by protein precipitation. Three hundred microliters of acetonitrile containing the internal standard (IS) ethylphosphonic acid at 10 μg/ml was added to 100 μl of broth. The samples were then mixed for 2 min and passed through a protein precipitation plate (Sirocco, Waters) using positive pressure. One volume of the supernatant was then mixed with one volume of 5 mM ammonium acetate, pH 4.8, and 5 μl was injected on an Agilent Zorbax Rapid Resolution High Definition (RRHD) HILIC Plus column (hydrophilic interaction chromatography column; 2.1 by 50 mm, 1.8-μm particle size). Chromatographic separation was achieved using 90:10 acetonitrile–50 mM ammonium acetate (pH 4.8) as mobile phase A and 50:40:10 acetonitrile–water–50 mM ammonium acetate (pH 4.8) as mobile phase B. Mobile phase A at 100% was kept for a minute, followed by an isocratic step at 100% mobile phase B for 2 min and 3 min of equilibration. A G6420A Agilent triple quadrupole mass spectrometer was employed for detection. The mass spectrometer was operated in multiple reaction monitoring (MRM) scan mode in negative polarity. The precursor ion for fosfomycin was *m/z* 137, and it was *m/z* 109 for the IS. The product ion for both was *m/z* 79. The source parameters were set as 3,500 V for capillary voltage, 350°C for gas temperature, and 40 lb/in^2^ for the nebulizer gas. All validation data were within the requirements of guidelines (20).

Amikacin concentrations were determined using an immunoassay technique according to the manufacturer's guidelines (Cobas Kit AMIK2; Roche Diagnostics) at the clinical laboratory of the Royal Liverpool Hospital, United Kingdom.
